# Supplementary material for: Effects of Elastic Resistance Exercise on Postoperative Outcomes Linked to the ICF Core Sets for Osteoarthritis after Total Knee Replacement in Overweight and Obese Older Women with Sarcopenia Risk: A Randomized Controlled Trial
Source: J Clin Med. 2020 Jul 11;9(7):2194. doi: 10.3390/jcm9072194 (PMC7408891; doi:10.3390/jcm9072194)
Supplement: Supplementary file 1 [file jcm-09-02194-s001.pdf]

Journal of
Clinical Medicine

Supplementary file

Table S1. Elastic resistance exercise.

Movement	Training volume (repetition × set)	Targeted muscle group	Duration (min)
**A. Warm-up**			
1. Mobility exercise of the neck, upper limbs, and back		Upper quarter flexors and extensors	5
2. Global flexion-extension of the lower limb		Lower quarter flexors and extensors	5
**B. Upper quarter**			
1. Seated chest press	10–20 × 3	Upper quarter extensors	5–10
2. Seated row	10–20 × 3	Upper quarter flexors	5–10
3. Seated shoulder press	10–20 × 3	Shoulder girdle muscle groups	5–10
**C. Lower quarter**			
1. Concentric–eccentric hip circumduction	10–20 × 3	Hip girdle muscle groups	5–10
2. Leg press	10–20 × 3	Lower quarter extensors	5–10
3. Leg curl	10–20 × 3	Lower quarter flexors	5–10
**D. Cool down**			5

Table 2. Exercise progression protocol.

	**Week**	**1**	**2**	**3**	**4**	**5**	**6**	**7**	**8**
Theraband color	Yellow	X	X						
	Red			X	X				
	Green					X	X		
	Blue							X	X
	Black								
	Silver								
Exercise loading	Repetition	10	20	10	20	10	20	10	20
	Set	3	3	3	3	3	3	3	3
	RPE[a]	13–14	14–15	13–14	14–15	13–14	14–15	13–14	14–15

X = the used Theraband color. [a]Ratings of perceived exertion according to the Borg sc

Table S3. Categories of the Brief ICF Core Set for osteoarthritis.

**ICF component**	**Chapter**	**ICF code**	**ICF category title**
**Body functions**	Sensory functions and pain	b280	Sensation of pain
	Neuromusculoskeletal and movement-related functions	b710	Mobility of joint functions
		b730	Muscle power functions
**Body structures**	Structures related to movement	s730	Structure of upper extremity
		s750	Structure of lower extremity
		s799	Structures related to movement, unspe
**Activities and participation**	Mobility	d445	Hand and arm use
		d450	Walking
	Self-care	d540	Dressing
**Environmental factors**	Products and technology	e115	Products and technology for personal living

		e150	Design, construction and building products and technology of buildings for public use	None identified
	Support and relationships	e310	Immediate family	None identified
	Services, systems and policies	e580	Health services, systems and policies	None identified

ROM, range of motion (degree); GS, gait speed (m/s); TCS, Timed chair stand (repetition); WOMAC−Pain, Western Ontario & McMaster Universities Osteoarthritis Index, pain subscale; WOMAC−PF, Western Ontario & McMaster Universities Osteoarthritis Index, physical difficulty subscale; AMI, appendicular mass index (kg/m^2). WOMAC-PF difficulty-ranked items: 9, Putting on socks; 11, Taking off socks.
